# Incomplete denitrification phenotypes in diverse *Thermus* species from diverse geothermal spring sediments and adjacent soils in southwest China

**DOI:** 10.1007/s00792-022-01272-1

**Published:** 2022-07-08

**Authors:** Chrisabelle C. Mefferd, Enmin Zhou, Cale O. Seymour, Noel A. Bernardo, Shreya Srivastava, Amanda J. Bengtson, Jian-Yu Jiao, Hailiang Dong, Wen-Jun Li, Brian P. Hedlund

**Affiliations:** 1 School of Life Sciences, University of Nevada, Las Vegas, Las Vegas, NV USA; 2 School of Earth Sciences, Yunnan University, Kunming, People’s Republic of China; 3 State Key Laboratory of Biocontrol, Guangdong Provincial Key Laboratory of Plant Resources and Southern Marine Science and Engineering Guangdong Laboratory (Zhuhai), School of Life Sciences, Sun Yat-Sen University, Guangzhou, People’s Republic of China; 4 Department of Geology and Environmental Earth Science, Miami University, Oxford, OH USA; 5 SWCA Environmental Consultants, Las Vegas, NV USA; 6 State Key Laboratory of Biogeology and Environmental Geology and Institute of Earth Sciences, China University of Geosciences, Beijing, People’s Republic of China; 7 Nevada Institute for Personalized Medicine, University of Nevada, Las Vegas, Las Vegas, NV USA

**Keywords:** Denitrification, *Thermus*, Nitrous oxide, Nitrate, Geothermal spring, Hot spring, Anaerobic respiration, Thermophiles

## Abstract

A few members of the bacterial genus *Thermus* have been shown to be incomplete denitrifiers, terminating with nitrite (NO_2_^−^) or nitrous oxide (N_2_O). However, the denitrification abilities of the genus as a whole remain poorly characterized. Here, we describe diverse denitrification phenotypes and genotypes of a collection of 24 strains representing ten species, all isolated from a variety of geothermal systems in China. Confirmed terminal products of nitrate reduction were nitrite or N_2_O, while nitric oxide (NO) was inferred as the terminal product in some strains. Most strains produced N_2_O; complete denitrification was not observed. Denitrification phenotypes were largely consistent with the presence of denitrification genes, and strains of the same species often had the same denitrification phenotypes and largely syntenous denitrification gene clusters. Genes for *nirS* and *nirK* coexisted in three *Thermus brockianus* and three *Thermus oshimai* genomes, which is a unique hallmark of some denitrifying *Thermus* strains and may be ecologically important. These results show that incomplete denitrification phenotypes are prominent, but variable, within and between *Thermus* species. The incomplete denitrification phenotypes described here suggest *Thermus* species may play important roles in consortial denitrification in high-temperature terrestrial biotopes where sufficient supply of oxidized inorganic nitrogen exists.

**Supplementary Information:**

The online version contains supplementary material available at 10.1007/s00792-022-01272-1.

## Introduction

Complete denitrification is the process by which microorganisms sequentially reduce nitrate (NO_3_^−^) to dinitrogen (N_2_) through anaerobic respiration using the following enzyme complexes: nitrate reductase, Nar and/or Nap (NO_3_^−^ → NO_2_^−^); nitrite reductase, NirS and/or NirK (NO_2_^−^ → NO); nitric oxide reductase, Nor (NO → N_2_O); and nitrous oxide reductase, Nos (N_2_O → N_2_). Traditionally, most denitrifiers were thought to be full denitrifiers that catalyze all steps of the pathway (Zumft 1997). However, microbes with truncated, incomplete, or alternative denitrification pathways are also known. For example, Sanford and colleagues (2012) described so-called atypical *nos* genes, comprising all known *nos* genes outside the *Pseudomonadota* (formerly informally Proteobacteria (Oren and Garrity, 2021)), and showed that they are frequently present in genomes that do not contain other denitrification genes. The atypical Nos is present in 13 bacterial phyla and both *Thermoproteota* and *Euryarchaeota*, and typically contains Sec, rather than Tat N-terminal signal sequences. Other denitrifiers appear to lack a cytochrome bc_1_ complex needed for N_2_O reduction to N_2_, but have an alternative complex III (ACIII), which performs the same function as the cytochrome bc_1_ complex by transferring electrons from the quinol pool to N_2_O (Refojo et al., 2012). Still others contain novel NO reductase systems, *sNOR*, *eNOR,* and *gNOR*, instead of the canonical *qNOR* or *cNOR,* which encode enzymes that reduce NO to N_2_O (Hemp & Gennis, 2008; Hemp et al., 2015; Stein et al., 2007). Finally, some microorganisms carry out incomplete denitrification ending with N_2_O (Hart et al., 1965), which can result from mutations in the *nosZ* gene (Zumft and Kroneck 2006) or the absence of *nos* genes (Murugapiran et al., 2013).

Terrestrial geothermal environments host an array of thermophiles with differing denitrification abilities. Various reductases have been found in sequenced genomes of thermophiles belonging to both *Thermoproteota* (e.g., *Aeropyrum*, *Sulfolobus*, and *Pyrobaculum*) and *Euryarchaeota* (e.g., *Archaeoglobus*) (Cabello et al. 2004); however, many of these have not been examined functionally. Nitrate reduction is better known in thermophilic members of several bacterial thermophiles, including members of the *Aquificota* (Hedlund et al. 2015a; Huber et al. 1992), *Bacillota* (formerly informally Firmicutes (Oren and Garrity 2021)) (Poli et al. 2009), and *Deinococcota*. Members of the bacterial genus *Thermus* are widely distributed in geothermal systems and have been studied as models of thermophilic nitrate reduction and denitrification (Cava et al. 2009). Some strains of *Thermus thermophilus* reduce nitrate to nitrite (Cava et al. 2008), while other isolates of *T. thermophilus* and *Thermus oshimai* are incomplete denitrifiers that terminate with N_2_O as a final product (Hedlund et al. 2011). However, most *Thermus* species have been shown to be positive for nitrate reduction using traditional biochemical tests (Albuquerque et al. 2018), which qualitatively measure nitrate and nitrite but not gaseous products. As a result, their capacity for denitrification is not well understood.

Several genetic and genomic studies have shed light on nitrate reduction and denitrification pathways in *Thermus*. Genes for nitrate reduction are found within the *nar* gene cluster and neighboring genes code for other denitrification genes (Gounder et al. 2011; Jiao et al. 2022; Mefferd et al. 2016; Murugapiran et al. 2013; Zhou et al. 2016). *Thermus* denitrification genes are sometimes present on plasmids (Ramirez-Arcos et al. 1998; Brüggemann et al. 2006). For example, genes encoding the ability to reduce nitrate to nitrite in *T. thermophilus* HB8 and NAR1 comprise three adjacent gene clusters, *nar*, *nrc*, and *dnr*, which are located on a megaplasmid termed the nitrate conjugative element (NCE). The megaplasmid carrying the NCE can be transferred among *T. thermophilus* strains by conjugation (Ramirez-Arcos et al*.* 1998), suggesting horizontal gene transfer (HGT) of denitrification genes is possible in natural populations of *Thermus*. Other denitrification genes can also be found on megaplasmids. Such is the case with *T. oshimai* JL-2 and *T. thermophilus* JL-18, whose megaplasmids are approximately 0.27 Mb and 0.26 Mb, respectively, and contain a gene cluster for the reduction of nitrate to N_2_O (Murugapiran et al., 2013). In some *Thermus* strains, the megaplasmid has been shown to be less stable and fast evolving compared to the chromosome (Brüggemann et al. 2006; Murugapiran et al. 2013). The possibility of HGT of denitrification genes is further supported by research done using *Thermus* species as models for thermophilic HGT. For instance, whole-genome studies of *Thermus scotoductus* SA-01 (Gounder et al. 2011) and *T. thermophilus* strains HB8 and HB27 (Kumwenda et al. 2014) have uncovered evidence for large-scale genetic loss, acquisition, and rearrangement. *T. thermophilus* HB27 is naturally competent, containing many proteins associated with competence and conjugation (Averhoff 2009), and can take up DNA at any stage during growth (Hidaka et al. 1994; César et al. 2011). Given the importance of HGT in the genus *Thermus* and the variable presence of denitrification genes on mobile genetic elements, denitrification genotypes and phenotypes might be expected to respond to selective pressures imparted by the geochemistry of individual springs and spring systems.

Our previous research on geothermal springs in the U.S. Great Basin has revealed the coexistence of *T. thermophilus*, *T. oshimai*, *T. aquaticus* and *Thermus sediminis* in sediments near the upper temperature limit for the genus (> 80 °C) (Dodsworth et al. 2011a; Hedlund et al. 2011; Zhou et al. 2020). The *T. thermophilus* and *T. oshimai* strains produced nitrous oxide as the terminal denitrification product and their presence coincided with high rates of nitrous oxide production in situ. Comparative analysis of representative genomes from these strains revealed a full complement of genes for the respiration of nitrate to nitrous oxide in each species, with genomes encoding the isofunctional tetraheme cytochrome *cd1*-containing nitrite reductase (NirS) and *T. oshimai* JL-2 additionally encoding the copper-containing nitrite reductase (NirK) (Murugapiran et al., 2013). Finally, in situ electron donor stimulation experiments showed that only yeast extract and peptone stimulated denitrification, whereas inorganic electron donors or defined organic electron donors (glucose and organic acid mixtures) had no effect, suggesting a key role for heterotrophs like *Thermus* in high-temperature denitrification (Dodsworth et al. 2011b).

These studies have shed some light on denitrification pathways in *Thermus* and inferred an important role for them in heterotrophic denitrification; however, they only encompass a few species and a limited geographic range, and do not provide a comprehensive characterization of the denitrification phenotypes across the genus. A recent paper describes the presence and evolution of denitrification gene clusters in representatives of most species of *Thermus*, but did not examine phenotypes (Jiao et al. 2022). The goal of this study was to characterize denitrification phenotypes in a diversity of *Thermus* species to gain insight into their potential roles in the nitrogen cycle in high-temperature environments. To address this goal, 24 strains representing ten *Thermus* species from various geothermal areas in China were grown under denitrifying conditions and nitrogenous products were measured. Additionally, denitrification genes were recovered from available genomes and by PCR using *Thermus*-specific primers designed in this study. These experiments show incomplete denitrification pathways to be common across the genus, further supporting their purported role as important incomplete denitrifiers in terrestrial geothermal systems.

## Materials and methods

### Isolation of Thermus strains

The sources of *Thermus* strains are described in Table 1. All strains were isolated by standard serial dilution plating technique onto R2A and T5 media (Ming et al. 2014) from geothermal spring sediments or mats or nearby geothermal soils in Yunnan Province, China, with the exception of *T. arciformis* JCM15153^T^, a type strain isolated from Guangxi Autonomous Region, China, which was included to increase taxonomic coverage. The physical and chemical properties of most springs that were sources for isolation of *Thermus* strains have been described previously (Hedlund et al. 2012; Hou et al. 2013; Song et al. 2013).

**Table 1:** *Thermus* strains and isolation source data

Strain	Isolation source	GPS coordinates	Spring temperature (°C) and pH	References
*T. amyloliquefaciens* YIM 77409^T^	Niujie Ancient Hot Spring	N 26.25033 E 99.98951	84 °C/7.4	Yu et al. (2015)
*T. amyloliquefaciens* YIM 77735-1	Soil sample near Shuirebaozha Spring	N 24.95002 E 98.43742	73 °C/7.0	This study
*T. antranikianii* 77311-1	Soil sample, near Shuirebaozha	N 24.95002 E 98.43742	73 °C/7.0	This study
*T. antranikianii* 77430-1	Soil sample near Shuirebaozha	N 24.95002 E 98.43742	73 °C/7.0	This study
*T. antranikianii* 77730	Soil sample near Shuirebaozha	N 24.95002 E 98.43742	73 °C/7.0	This study
*T. arciformis* JCM 15153^T^	Hot Spring in Laibin, Guangxi	N 23.97111 E 109.75472	N/A	Zhang et al. (2010)
*T. brockianus* YIM 77420-2	Niujie Ancient Hot Spring	N 26.25033 E 99.98951	84 °C/7.4	Yu et al. (2015)
*T. brockianus* YIM 77904	Gongxiaoshe Spring	N 25.44012 E 98.44081	73.8 °C/7.29	This study
*T. brockianus* YIM 77927	Hehua Spring	N 23.65489 E 97.87011	73 °C/7.0	This study
*T. brockianus* YIM 79134	Shuirebaozha Spring	N 24.95014 E 98.43743	79.8 °C/7.5	Ming et al. (2014)
*T. caliditerrae* YIM 77925^T^	Shuirebaozha Spring	N 24.95014 E 98.43743	79.8 °C/7.5	Ming et al. (2014)
*T. igniterrae* YIM 77777-1	Gongxiaoshe Spring	N 25.44012 E 98.44081	73.8 °C/7.29	This study
*T. oshimai* YIM 77359	Hamazui Spring	N 24.95351 E 98.43819	64 °C/8.0	This study
*T. oshimai* YIM 77838-1	Xianrendong Spring	N 25.46721 E 98.49097	73 °C/8.0	This study
*T. oshimai* YIM 77923-2	Hehua Spring	N 23.65489 E 97.87011	73 °C/7.0	This study
*T. scotoductus* YIM 77445-2	Hamazui Spring	N 24.95351 E 98.43819	64 °C/8.0	This study
*T. tengchongensis* YIM 77357	Shuirebaozha Spring	N 24.95002 E 98.43742	70 °C/8.0	This study
*T. tengchongensis* YIM 77392	Shuirebaozha Spring	N 24.95002 E 98.43742	70 °C/8.0	This study
*T. tengchongensis* YIM 77392-1	Shuirebaozha Spring	N 24.95002 E 98.43742	70 °C/8.0	This study
*T. tengchongensis* YIM 77401	Hamazui Spring	N 24.95351 E 98.4382	85 °C/8.0	This study
*T. tengchongensis* YIM 77410	Hamazui Spring	N 24.95351 E 98.43819	85 °C/8.0	This study
*T. tengchongensis* YIM 77727	Hamazui Spring	N 24.95351 E 98.43819	85 °C/8.0	This study
*T. tengchongensis* YIM 77924^T^	Soil sample near Shuirebaozha Spring	N 24.95002 E 98.43742	82 °C/7.5	Yu et al. (2013)
*T. thermophilus* YIM 77430-2	Soil sample near Shuirebaozha Spring	N 24.95002 E 98.43742	73 °C/7.0	This study

*N/A* not available

### Screen for nitrate reduction phenotype

*Thermus* strains were revived from frozen stocks on Castenholz Medium D (CMD) agar plates amended with 9 mM nitrate and supplemented with 0.1% yeast extract and 0.1% tryptone and adjusted to pH 8.2 (Castenholz 1969; Hedlund et al. 2011). *Thermus* strains were screened for nitrate reduction phenotype by testing for the ability to grow in anaerobic liquid CMD modified as described above, but with 4.5 mM nitrate. The anaerobic medium was sparged with N_2_ for 45 min to remove oxygen and distributed into glass serum bottles, or Balch tubes with Durham vials, in an anaerobic chamber (Coy Type B, Coy Laboratory Products Inc., Grass Lake, MI, USA) containing an atmosphere of N_2_ (~ 90%), CO_2_ (~ 5%), and H_2_ (~ 5%). The culture bottles were sealed with butyl rubber stoppers and aluminum crimps and the headspace was exchanged prior to autoclaving by 5 cycles of evacuation (30 s) and filling to 1 atm with 99.999% He.

For all nitrate reduction experiments, a pure colony of each strain was suspended and grown in 10 mL of anaerobic medium in 25 mL glass serum bottles to serve as a starter culture. To dilute contaminating N_2_ and ensure denitrification pathways were active, cells were grown to early stationary phase and passed using He-rinsed syringes with a 1:50 inoculum into pre-warmed medium twice before a final transfer into experimental bottles. 160 mL serum bottles containing 40 mL of liquid medium described above, or Balch tubes with Durham vials with 10 mL of liquid medium, were used for the final transfer. Serum bottles were incubated in the dark at 60 °C with rotary shaking at 100 rpm with serum bottles in a horizontal position to maximize gas equilibration. Balch tubes were incubated in the dark at 60 °C in a static incubator. Unless otherwise noted, cell density was measured using a Petroff–Hausser counting chamber on an Olympus BX-51 phase-contrast microscope with brightness and contrast optimized using PictureFrame software (Optronics, Goleta, CA, USA).

Nitrate, nitrite, N_2_O, and N_2_ were then assayed after 96 h using the methods described below. All strains were capable of nitrate reduction and robust growth in the medium used for this work. A few strains initially included in the study, *T. brockianus* YIM 77709, *T. scotoductus* YIM 77398, and *T. tengchongensis* YIM 77427, did not grow well under the denitrification conditions tested here and were subsequently dropped from the study, leaving 24 strains. *T. calditerrae* YIM 77777 failed to grow under denitrification conditions, consistent with the absence of annotated denitrification genes in its genome (Mefferd et al. 2016). All strains were tested in triplicate and data presented are from replicates with a final cell concentration of ≥ 1 × 10^6^ cells/mL.

### Detection of terminal denitrification nitrogen products

To measure aqueous nitrate and nitrite concentrations, approximately 6 mL of liquid medium was sampled after a 96 h endpoint, filtered through a 0.2 μm filter (28145-501 VWR, Radnor, PA, USA), stored at 4 °C, and analyzed by colorimetric methods and confirmed using ion chromatography (IC). Nitrite concentrations were measured by diazotization with sulfanilamide, followed by coupling with *N*-(1-naphthyl)-ethylenediamine dihydrochloride (LaMotte, Chestertown, MD, USA). For nitrate measurements, powdered cadmium was used to reduce nitrate to nitrite prior to diazotization (LaMotte, Chestertown, MD, USA). To confirm colorimetric measurements, IC analysis was performed on samples from a subset of experiments as previously described (Hou et al. 2013) using a Dionex DX-500 Chromatograph with an AS22 anion exchange column with a 4.5 mM Na_2_CO_3_/0.8 mM NaHCO_3_ eluent. To measure N_2_O, headspace gas samples were collected from culture bottles at a 96 h endpoint for gas chromatography (C2014 Shimadzu GC) analysis. Headspace N_2_O concentration was measured by injecting a 2 mL headspace gas sample into a GC-2014 Nitrous Oxide Analyzer (Shimadzu, Moorpark, CA, USA) operated as described previously (Dodsworth et al. 2011a, b). To detect N_2_ gas, cultures were screened for the ability produce N_2_ gas in Durham vials in 25 mL Balch tubes after 96 h and by gas chromatography using a thermal conductivity detector (TCD) (Dodsworth et al. 2011a). Statistical significance for all measurements was calculated by comparing data to an uninoculated control using a Student’s *t* test in R (*p* < 0.1).

### DNA extraction, PCR amplification of 16S rRNA genes, and DNA sequencing

DNA was extracted from *Thermus* cell pellets using the FastDNA Spin Kit for Soil (MP Biomedicals) according to the manufacturer’s protocol. 16S rRNA genes were amplified with PCR using primers specific for bacteria: 9bF (Eder et al. 1999) and 1512uR (Eder and Huber 2002). The 25 μL PCR mixture contained 10–125 ng of DNA, 200 nM of each primer, 200 μM each dNTP (Promega, Madison, WI, USA), 1.5 mM MgCl_2_, 0.625 U of GoTaq DNA polymerase (Promega), and 1 × GoTaq buffer (Promega). Cycling conditions were as follows: denaturation at 95 °C for 4 min followed by 30 cycles of denaturation (30 s at 95 °C), annealing (1 min at 55 °C), and elongation (2 min at 72 °C), with a final elongation step (7 min at 72 °C). PCR products were sequenced using the Sanger method at Functional Biosciences, Madison, WI, using the forward and reverse PCR primer.

### Design of PCR primers and amplification of nitrogen-oxide reductase genes

Conserved regions for *narG*, *nirK*, *nirS*, and *norB* in *Thermus* species were chosen to design the primers used in this study. Existing primers for nitrogen-oxide reductases (Braker et al. 1998; Phillippot et al. 2002; Throbäck et al. 2004) were shown or predicted to be ineffective with *Thermus* genes (data not shown). *Thermus* denitrification gene sequences were harvested from genomes (Gounder et al. 2011; Mefferd et al. 2016; Murugapiran et al. 2013; Zhou et al. 2016) available at the Joint Genome Institute’s Integrated Microbial Genomes (IMG) website (Markowitz et al. 2014) and RAST (Aziz et al. 2008; Overbeek et al. 2014) and used for alignment. For each gene, the available sequences were aligned using default parameters using MUSCLE (Edgar 2004; Dereeper et al. 2008). Conserved regions used for primer design were chosen manually and were predicted to give PCR products of ~ 1000 bp and include conserved functional domains.

To optimize annealing temperatures for each primer set, gradient PCR amplification over a range of ± 5 °C from the mean of the calculated melting temperature for each primer set. Primer combinations and optimal annealing temperatures can be found in Table S2. The sequences and locations of the binding sites of the primers are shown in Table 1.

Hot-start PCR for amplification of *narG*, *nirK*, *nirS*, and *norB* was performed with DNA from each *Thermus* strain as template. The 25 μL PCR mixture contained 10–125 ng DNA, 200 nM of each primer, 1.5 mM MgCl_2_, 200 μM each dNTP, 0.625 U of GoTaq DNA Polymerase (Promega, Madison, WI), and 1 × Green GoTaq Reaction Buffer. Cycling conditions were as follows: denaturation at 95 °C for 4 min followed by 35 cycles of denaturation (2.5 min at 95 °C), annealing (1 min, see Table S2 for temperatures), and elongation (2.5 min at 72 °C), with a final elongation step at 72 °C for 7 min. PCR products were sequenced using the Sanger method at Functional Biosciences, Madison, WI, using the forward and reverse PCR primer.

### Phylogenetic analysis and tree construction

To determine the evolutionary relationships among *Thermus* species, a phylogenetic tree was generated from the 16S rRNA gene sequences of the isolates. 16S rRNA genes of the new isolates and all type strains of the genus *Thermus* were aligned using the mothur-provided SILVA alignment (Quast et al. 2012) in mothur v.1.39.5 (Schloss et al. 2009). The alignment was checked manually and filtered using the mothur-provided, SILVA-compatible 1349-position Lane mask (Lane 1991). Phylogenetic trees were constructed using a maximum-likelihood algorithms within RAxML (Stamatakis et al. 2014) using the TIM3-F + 1 + G4 model, chosen based on the Bayesian Information Criterion (BIC). Neighbour-Joining and Maximum-parsimony trees were also implemented within MEGA 7.0 (Kumar et al. 2016). Branch support values were based on bootstrap analysis performed using 1000 replicates.

### Nucleotide accession numbers

Nucleotide accession numbers for all gene fragments amplified by PCR are available in GenBank under the following accession numbers: 16S rRNA genes (ON429762-ON429782) and denitrification genes (ON456559-ON456600) are available in GenBank. Accession numbers for genome sequences are provided in Table 2.

**Table 2:** Summary of denitrification phenotype and genotype

Strain	Reduction of			Genes present (genome accession number)
	Nitrate	Nitrite	Nitric oxide	
*T. amyloliquefaciens* YIM 77409^T^	+	(+)	+	*narG, nirS, norB* (GCA_000744885.1)
*T. amyloliquefaciens* YIM 77735-1	+	−	−	*narG, norB*
*T. antranikianii* YIM 77311-1	+	(+)^a^	+ ^a^	*narG*
*T. antranikianii* YIM 77430-1	+	(+)	+ ^a^	*narG, nirK*
*T. antranikianii* YIM 77730	+	(+)	+ ^a^	*narG, nirK*
*T. arciformis* JCM 15153^T^	+	(+)	+	*narG, nirS, norB* (GCA_900102145.1)
*T. brockianus* YIM 77420-2	+	(+)	−	*narG, nirS*
*T. brockianus* YIM 77904	+	(+)	−	*narG, nirK, nirS*
*T. brockianus* YIM 77927	+	(+)	−	*narG*, *nirK*, *nirS* (GCA_021462505.1)
*T. brockianus* YIM 79134	+	(+)	+ ^a^	*narG, nirK, nirS*
*T. caliditerrae* YIM 77925^T^	+	−	−	*narG, nirS* pseudogene*, norB* pseudogene (GCA_021462525.1)
*T. igniterrae* YIM 77777-1	+	−	−	*narG*
*T. oshimai* YIM 77359	+	(+)	+	*narG, nirK, nirS, norB*
*T. oshimai* YIM 77838-1	+	(+)	-^b^	*narG, nirK, nirS, norB*
*T. oshimai* YIM 77923-2	+	(+)	−	*narG, nirK, nirS*
*T. scotoductus* YIM 77445-2	+	−	−	*narG*
*T. tengchongensis* YIM 77357	+	(+)	+	*narG, nirS, norB*
*T. tengchongensis* YIM 77392	+	(+)	+	*narG, nirS, norB*
*T. tengchongensis* YIM 77392.1	+	(+)^a^	+	*narG, norB*
*T. tengchongensis* YIM 77401	+	(+)	[ +]	*narG, nirS, norB* (GCA_000744175.1)
*T. tengchongensis* YIM 77410	+	(+)	+	*narG, nirS, norB*
*T. tengchongensis* YIM 77727	+	(+)^a^	+	*narG, norB*
*T. tengchongensis* YIM 77924^T^	+	(+)	+	*narG, nirS, norB* (GCA_021462405.1)
*T. thermophilus* YIM 77430-2	+ ^a^	−	−	*nirS, norB*

+ Statistically significant decrease in substrate and/or increase in product compared to an uninoculated negative control (*p* ≤ 0.1)

(+)Inferred nitrite reduction activity due to a significant decrease in nitrite or significant increase in N_2_O compared to negative control (*p* ≤ 0.1)

[+]Incomplete denitrifier that produces ≥ 1 mM of N_2_O in at least two replicates in multiple assays

− Phenotype not detected

^a^Phenotype detected, but no genome was available and PCR for the corresponding gene was negative

^b^Denitrification genes present, but phenotype not detected

^c^Unless a genome accession is included, genes were amplified by PCR and sequenced

## Results

### Isolation and identification of a diverse collection of Thermus strains from geothermal springs in Yunnan, China

**Fig. 1:**
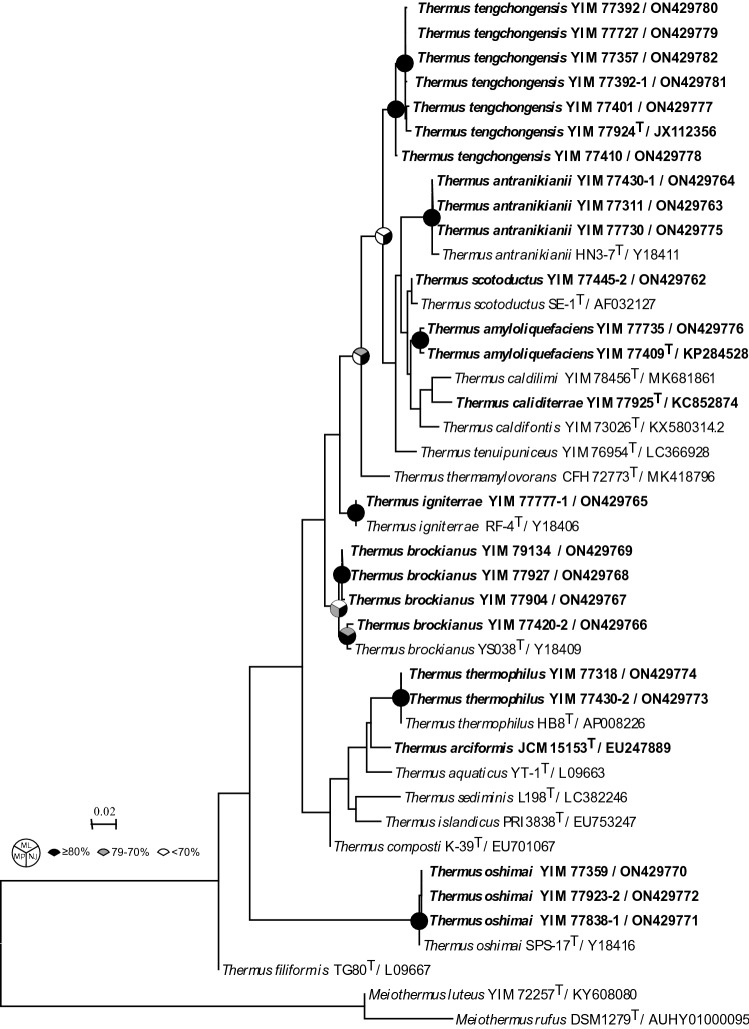
16S rRNA gene phylogeny. Maximum-likelihood phylogeny based on 16S rRNA gene sequences of all type strains of *Thermus* plus the isolates described in this manuscript (in bold). Shading at nodes depict bootstrap support using maximum-likelihood, neighbor-joining, and maximum parsimony

*Thermus* strains were isolated by serial dilution and plating from samples collected from sediments and mats from springs and heated soils at Rehai National Park, Gongxiaoshe Spring, Hehua Spring, and Nuijiea Ancient Hot Spring, all in Yunnan Province, China (Table 1). 16S rRNA gene sequencing and phylogenetic analysis revealed that the strains belonged to nine different species: *Thermus amyloliquefaciens, Thermus antranikianii*, *Thermus brockianus*, *Thermus calditerrae*, *Thermus igniterrae*, *T. oshimai*, *T. thermophilus*, *T. scotoductus*, and *Thermus tengchongensis*, spanning about half of the *Thermus* species with validly published names (Fig. 1) and most species known to be capable of nitrate reduction or denitrification.

### Determination of nitrate reduction intermediates and terminal products

The newly isolated *Thermus* strains, plus *Thermus arciformis* JCM 15153^T^, were screened for growth under anaerobic conditions with nitrate as the sole terminal electron acceptor. Almost all strains grew well in anaerobic media with nitrate as the sole terminal electron acceptor and were inferred denitrifiers since fermentation has not been described in *Thermus* (Albuquerque et al. 2018). *T. caliditerrae* YIM 77,777 did not reduce nitrate, as confirmed by the absence of denitrification genes in its genome (Mefferd et al. 2016), and was dropped from the study.

The 24 strains reduced nitrate but failed to produce N_2_. Terminal denitrification products varied among the isolates (Table 2); however, most species were coherent with regard to denitrification phenotype. Most strains (*n* = 11) produced N_2_O as the final denitrification product, including all strains of *T. antranikianii* (*n* = 3) and *T. tengchongensis* (*n* = 7) and the single strain of *T. arciformis*. By comparison, several strains only reduced nitrate to nitrite, including the single tested strains of *T. caliditerrae*, *T. igniterrae, T. scotoductus,* and *T. thermophilus*. However, consistent denitrification phenotypes were not observed in *T. amyloliquefaciens*, *T. brockianus,* or *T. oshimai*, where different strains terminated with different intermediates of denitrification (Table 2). In* T. amyloliquefaciens*, one strain reduced nitrate to N_2_O, whereas the other only reduced nitrate to nitrite. In three of the four *T. brockianus* strains, nitrate was removed during anaerobic growth, yet neither nitrite, N_2_O, nor N_2_ were detected at the 96 h endpoint that was assayed. This phenotype points to NO as the most likely denitrification product in these strains, which was also supported by the detection of *narGHI*, *nirS*, and *nirK* genes in the genome of *T. brockianus* YIM 77927, which lacks *norCBH* genes (Fig. 3). A similar genotype and phenotype was observed for *T. oshimai* YIM 77923-2, and similar genotypes (*nar* and *nir* genes but lacking *nor* genes) have been observed previously in genomes from *T. antranikianii* DSM 12462^T^, *Thermus parrtaviensis* RL^T^, *Thermus thermamylovorans* CFH72773^T^, *Caldithermus terrae* DSM 26712^T^, and *Caldithermus chliarophilus* DSM 9957^T^ (Jiao et al. 2022). These genomes also lack *nrf* systems that could support dissimilatory nitrite reduction to ammonium as an alternative pathway. The possible accumulation of NO as the main denitrification product by many members of the *Thermaceae* is somewhat unexpected due to the toxicity of NO, so this possibility should be examined experimentally. The other strains of *T. brockianus* (YIM 779134) and *T. oshimai* (YIM 77359) produced N_2_O as the terminal product.

**Fig. 2:**
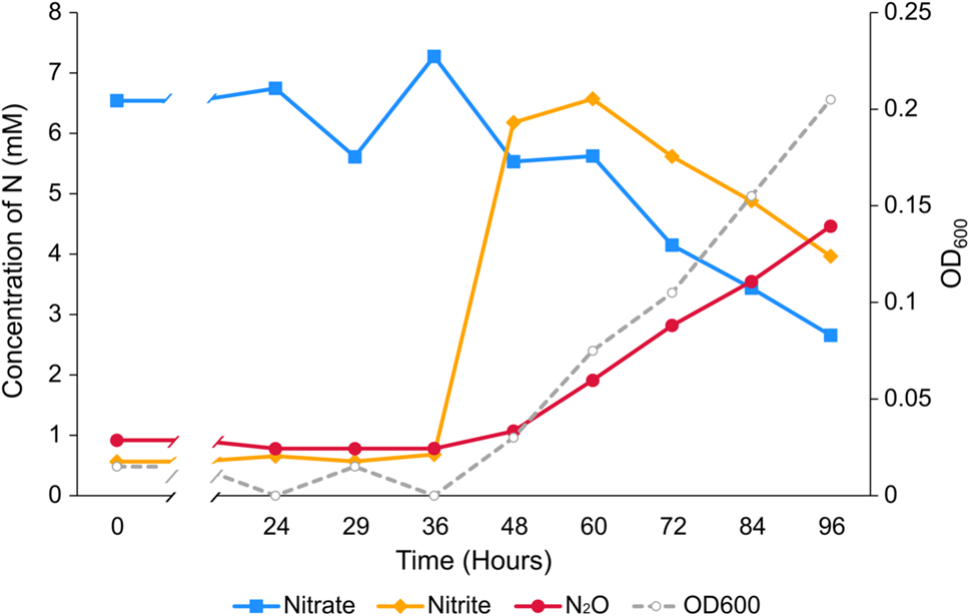
Near-stoichiometric conversion of nitrate to N_2_O during growth of *T. arciformis* JCM 15153^T^. *T. arciformis* JCM 15153^T^ cells were grown with 9 mM NO_3_^−^-amended CMD and sampled periodically for quantification of cell density using Spectrophotometry and possible denitrification intermediates (i.e., nitrite and N_2_O). Reduction of nitrate to N_2_ was never detected in Balch tubes with Durham vials or by GC-TCD. Data are representative of triplicate experiments

To examine a nitrate reduction phenotype in more detail, *T. arciformis* JCM15153^T^ was selected for further analysis based on its consistent growth phenotype (Fig. 2). There was a long lag phase until exponential growth was detected after 36 h after which a near-stoichiometric conversion of nitrate to N_2_O was observed, with transient production of nitrite and no production of N_2_.

### Presence and arrangement of denitrification gene clusters

**Fig. 3:**
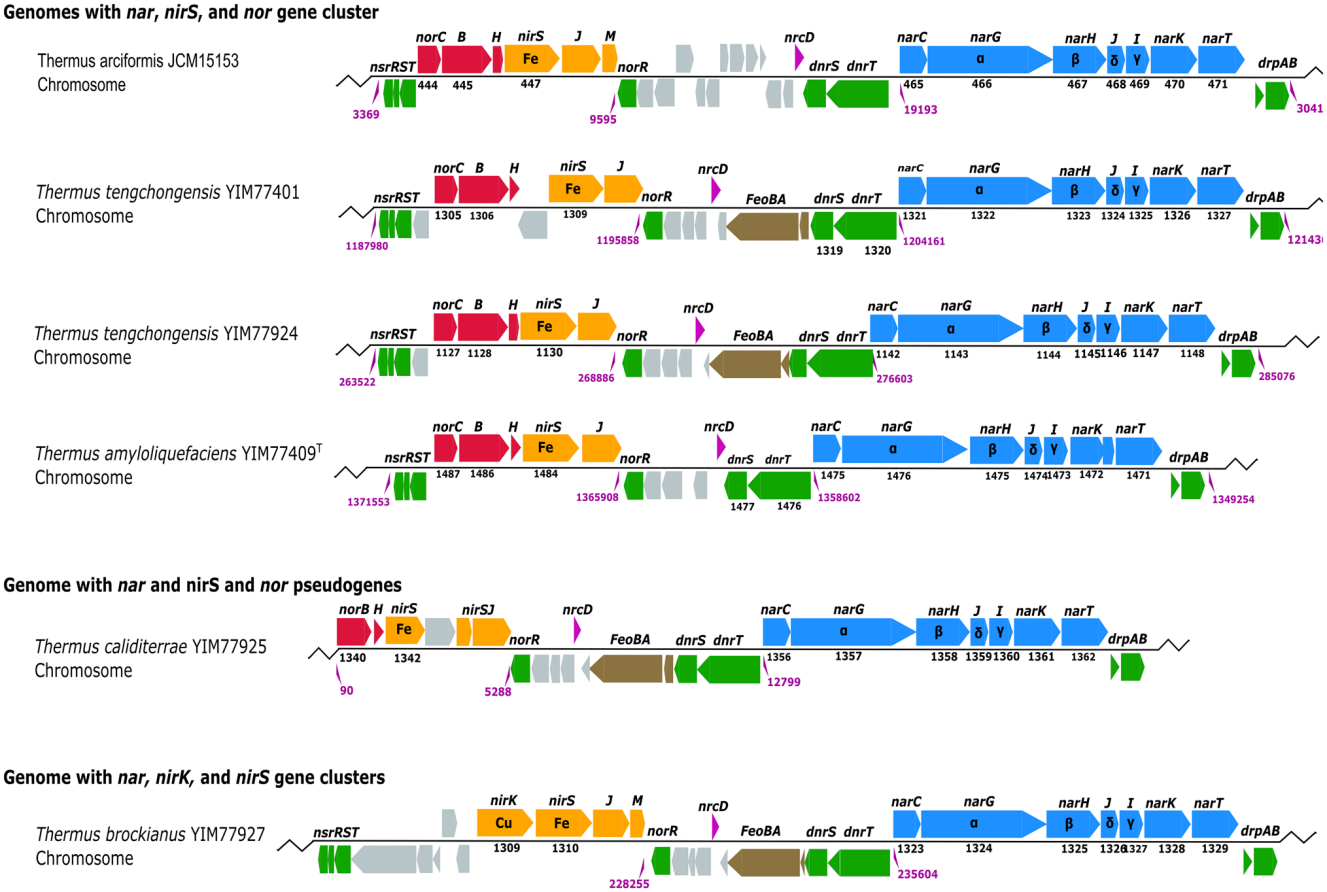
Denitrification gene clusters. *nar* operon and neighboring genes involved in denitrification located on the chromosome of *T. amyloliquefaciens* YIM77409^T^, *T. arciformis* JCM 15153^T^. *T. brockianus* YIM 77927, *T. caliditerrae* YIM 77925^T^, *T. tengchongensis* YIM 77401, and *T. tengchongensis* YIM 77924^T^. Numbers below selected genes indicate provisional ORF numbers in IMG for *T. tengchongensis* YIM 77401 (BS84DRAFT_1309) and *T. amyloliquefaciens* YIM 77409^T^ (BS74DRAFT_1484), and RAST. Selected locations in the chromosome are indicated below in purple text. Annotated catalytic and structural proteins: *nar* nitrate reductase; *nir* nitrite reductase; *nor* nitric oxide reductase; *nrcD* ferredoxin associated with denitrification gene cluster in *Thermus* (Cava et al. 2004). Annotated regulatory proteins: *dnr* denitrification regulator; *nsr* NO-dependent regulator of *nir/nor* (Alvarez et al., 2017); *drp* nitrate sensor proteins (Chahlafi et al. 2018); *norR* MarR-family transcription factor conserved in *Thermus* denitrification gene clusters (Sánchez-Costa et al. 2020). Other annotated genes: *Feo* ferrous iron transport system

Denitrification genes were recovered from newly sequenced genomes of *T. arciformis* JCM15153^T^, *T. brockianus* YIM 77927, *T. caliditerrae* YIM 77925^T^, and *T. tengchongensis* YIM 77924^T^, and previously available genomes of *T. amyloliquefaciens* YIM 77409^T^ (Zhou et al. 2016) and *T. tengchongensis* YIM 77401 (Mefferd et al. 2016). The genome of *T. caliditerrae* YIM 77777 did not contain denitrification genes (Mefferd et al. 2016), consistent with its inability to grow in nitrate reduction experiments. The other six strains contained a complete nitrate reductase operon (*narGHJI*) and two nitrate/nitrite transporters (*narK1* and *narK2*). Genes encoding nitrite reductase (*nirS*) and cytochrome c-dependent nitric oxide reductase (*norBCH*) were also found in proximity to the nitrate reductase operon, except for strain *T. brockianus* YIM 77927, which contained two nitrite reductases (*nirK* and *nirS*) and was missing *nor* genes. Genes encoding NorR, the MarR-family transcription factor conserved in *Thermus* denitrification gene clusters (Sánchez-Costa et al. 2020), was conserved and syntenic in all genomes. As noted previously (Murugapiran et al. 2013; Zhou et al. 2016; Mefferd et al. 2016), nitrous oxide reductase (*nos*) genes were absent in all *Thermus* genomes examined. Denitrification genes in these six genomes, plus *T. scotoductus* SA-01 (Gounder et al. 2011), *T. thermophilus* JL-18, and *T. oshimai* JL-2 (Murugapiran et al. 2013), were co-localized and spanned over 25 kbp (Fig. 3). The genes were generally syntenous, but with some rearrangements among *norCBH*, *nirK*, and *nirS*. The (*narGHJIK(K2)*) operons were entirely syntenous and consistently divergent from the two component regulatory system, *dnrST* (Alvarez et al. 2017). Genes encoding the *drpAB* nitrate sensor protein system (Chahlafi et al. 2018) were consistently downstream of the *nar* operon. Genes encoding *nrcD*, a ferredoxin associated with denitrification gene clusters in *Thermus* (Cava et al. 2004) were conserved and syntenous. The denitrification gene cluster was chromosomally encoded on each of the six newly sequenced genomes.

PCR was used to obtain denitrification genes from the other strains. Since existing primers (Braker et al. 1998; Dodsworth et al. 2011a; Phillippot et al. 2002; Throbäck et al. 2004) were shown or predicted to be ineffective for all *Thermus* denitrification genes, a few sets of novel primers were designed and optimized based on existing *Thermus* denitrification genes (Tables S1, S2). All PCR amplicons were most closely related to those from other *Thermus* species, with nucleotide sequence identities of 82–99%. Putative *narG* fragments were amplified from DNA extracts of all *Thermus* strains tested, except *T. thermophilus* YIM 77430-2, despite its ability to grow under nitrate-reducing conditions and produce nitrite as a terminal product (Table 2). The presence of *nir* and *nor* genes was variable. Including genome-derived genes and PCR products, genes encoding the isoenzymes NirK (*n* = 9) and NirS (*n* = 14) were both common in *Thermus* genomes. Two strains of *T. antranikianii* appeared to have only *nirK*. Others appeared to have only *nirS* (*n* = 9), including most members of *T. tengchongensis* (*n* = 5), one strain each of *T. brockianus* and *T. amyloliquefaciens*, and the only strain of *T. caliditerrae*. Other species harbored both *nirK* and *nirS*, including all three strains of *T. oshimai* and three of four strains of *T. brockianus*. Including both genome- and PCR-derived data, *norB* was present in at least 14 of the genomes, including all 8 isolates of *T. tengchongensis*, both strains of *T. amyloliquefaciens*, two of three strains of *T. oshimai* and the only strains of *T. arciformis*, *T. calditerrae*, *T. thermophilus*, and *T. scotoductus*.

In general, denitrification genes appeared to be intact, but it should be noted that pseudogenes predicted to encode proteins with C-terminal truncations in NirS and NorB were annotated in *T. caliditerrae* YIM 77925^T^. This genome is also missing *norC*, an essential periplasmic Nor subunit that shuttles electrons to the catalytic subunit NorB (Thorndycroft et al. 2007; Hino et al. 2010). Together, this is consistent with the observation that nitrite was the terminal product in *T. caliditerrae* YIM 77925^T^ cultures (Table 2). Interestingly, there is no indication that truncations in NirS and NorB include binding sites or amino acid residues of known functional importance (Rees et al. 1997; Watmough et al. 1999; Hemp and Gennis 2008).

The presence of denitrification genes in the *Thermus* strains studied here was generally consistent with the denitrification phenotypes; however, in several cases the detected phenotype and genotype did not agree (Table 2). *nar* genes were not found in *T. thermophilus* YIM 77430-2 using primers for this study, but nitrate reductase activity was detected. *nir* genes were not detected for *T. antranikianii* YIM 77311 and *T. tengchongensis* YIM 77392, though there was measured nitrite reductase activity. Finally, *nor* genes were not detected in any *T. antranikianii* strains or *T. brockianus* YIM 79134, though N_2_O was detected. In these cases, inconsistencies are likely the result of the limited sensitivity of the PCR primers used in this work. Future work will be needed to design primers with better coverage within the *Thermales*, perhaps using primer sequences similar to those described here but with additional degeneracies, or by incorporating alignments with denitrification gene sequences from other published genomes.

## Discussion

Here, 24 strains representing nine different *Thermus* species isolated from diverse geothermal areas in China were shown to be capable of respiratory nitrate reduction or incomplete denitrification. Combined with previous studies of incomplete denitrification in *Thermus* from other locations, this work shows that incomplete denitrification pathways are common across species of the genus *Thermus* regardless of geographic location. Consistent with the incomplete denitrification phenotypes of other *Thermus* strains, nitrate reduction to either nitrite, NO, or N_2_O as terminal products indicates varying denitrification capabilities within *Thermus*, with most strains ending with N_2_O as a final product. These data support the inference that *Thermus* in geothermal areas may serve as a source of N_2_O, a strong greenhouse gas and stratospheric reactant, as has been measured in geothermal springs in the U.S. Great Basin (Hedlund et al. 2011). Incomplete denitrification pathways producing N_2_O may also promote N-cycling within hot spring systems because N_2_O is more soluble than N_2_ and its reduction can be coupled to proton translocation and growth (Flock et al. 2005; Zumft and Kroneck 2006). In contrast, N_2_ has a lower aqueous solubility and is relatively inert, especially in systems with sufficient dissolved inorganic nitrogen supply where selection for or expression of nitrogen fixation may be weak. However, single-cell genomes and metagenome-assembled genomes from members of the *Caldarchaeales* (synonym: 'Aigarchaeota’) show several members of this often-abundant, but not isolated group to contain putative N_2_O reductase genes (Rinke et al. 2013; Hedlund et al. 2015b) and no other denitrification genes and are, therefore, possible metabolic partners of *Thermus*. Similarly, multiple species of *Thermoflexus* that often cohabit with *Thermus* have putative N_2_O reductase genes, yet this function was not demonstrated in cultures of *Thermoflexus hugenholtzii* (Thomas et al. 2021). A multispecies coupled denitrification pathway is consistent with high rates of complete denitrification in sediments in Great Boiling Spring, NV, inferred from much higher N_2_O fluxes in acetylene block experiments compared to no-acetylene controls (Dodsworth et al. 2011b; Hedlund et al. 2011). In contrast, similar rates of nitrous oxide production were obtained with and without acetylene blocks in heated soils adjacent to springs in Yellowstone National Park, inferring high rates of N_2_O production and low rates of complete denitrification (Burr et al. 2005). Whether other high-temperature geothermal systems support both N_2_O emissions and complete denitrification has yet to be addressed. It is similarly unknown whether any *Thermus* strains are capable of complete denitrification or of the terminal step of denitrification at all. At least one report inferred complete denitrification in *T. thermophilus* HB27 despite the absence of an annotated nitrous oxide reductase, based on missing nitrogen balance during denitrification experiments (Bricio et al. 2011), though we recommend this result should be followed up more rigorously, for example, using stable isotopes of nitrogen followed by mass spectrometry.

The synteny of denitrification gene clusters in the diverse *Thermus* isolates suggests these gene clusters evolve largely together, whether vertically or horizontally. Several of the *Thermus* strains studied here contain a *nirK* homolog (two *T. antranikianii* strains, three *T. brockianus* strains, and three *T. oshimai* strains) and were capable of nitrite reduction, inferring activity for the NO-forming, Cu-containing nitrite reductase in denitrification. Alternatively, or in addition, several strains contained homologs of the isofunctional tetraheme cytochrome *cd1*-containing nitrite reductase, *nirS* (*T. amyloliquefaciens* YIM 77409^T^, *T. arciformis* JCM 15153^T^, three *T. brockianus* strains, three *T. oshimai* strains, and five *T. tengchongensis* strains). Six of the strains belonging to *T. brockianus* and *T. oshimai* encoded both NirK and NirS homologs. NirK and NirS were previously considered incompatible (Zumft 1997) but several species of *Thermus* have been previously noted to encode both enzymes, including strains of *T. scotoductus* (Gounder et al. 2011), *T. oshimai* (Murugapiran et al. 2013) and *T. antranikianii* (Liu et al. 2020). The current study adds *T. brockianus* to that list. Studies in *T. antranikianii* DSM 12462^T^ (Liu et al., 2020) showed that both genes are co-transcribed during denitrification, with higher expression of *nirK* under a wider range of nitrite concentrations and only *nirS* expressed in the presence of oxygen. *Thermus* strains encoding both nitrite reductases have also been shown to lower the transient and final concentrations of nitrite during the denitrification with nitrate as the substrate, inferring more efficient use of available nitrite under both high and low substrate concentrations (Hedlund et al. 2011). The co-occurrence of these nitrite reductases in several species and the lack of nitrous oxide reductase across the genus appear to be unique features of the genus *Thermus* and further underscores the likely ecological importance of *Thermus* in denitrification.

This study did not address the distribution of *Thermus* genotypes and phenotypes across physicochemical space. Thus, although it is reasonable to expect physicochemical conditions and ecological relationships to constrain the presence and composition of denitrification pathways, it is currently unknown how *Thermus* denitrification pathways are distributed in physicochemical space in geothermal systems. Temperature influences both steps of the oxidative nitrogen cycle in terrestrial geothermal systems (De la Torre et al. 2008; Edwards et al. 2013; Dodsworth et al. 2011a; Spieck et al. 2020), which in turn influences the supply of oxidized nitrogen in systems sourced by ammonium, which is commonly the dominant form of dissolved inorganic nitrogen in geothermal systems (Holloway et al. 2011). Similarly, as these enzymes use cofactors containing different trace metals such as molybdenum, iron, and copper, the availability of these metals may constrain these organisms and pathways. For example, Nar and NirK use molybdenum and copper as cofactors, which are both less soluble in the presence of hydrogen sulfide (Anbar and Knoll 2002). Given the likely importance of *Thermus* to the high-temperature nitrogen cycle, the distribution of *Thermus* species and denitrification genes within the greater context of denitrification genotypes is ripe for further study and could be addressed using coupled metagenomics and physicochemical data.

## Conclusions

Incomplete denitrification pathways are common in the genus *Thermus*. More specifically, *Thermus* denitrification is characterized by nitrate reduction to nitrite, NO, or N_2_O as terminal products, evident by the varying phenotypes and genotypes. Despite a few inconsistencies between detected reductase activity and amplified genes, the work done here significantly expands on the current state of knowledge of denitrification in *Thermus* and further suggests and important role for the genus in thermophilic denitrification in terrestrial geothermal environments.

## Supplementary Information

Below is the link to the electronic supplementary material.

Supplementary file1 (DOCX 16 KB)

Supplementary file2 (DOCX 14 KB)
