# Supplementary material for: Incomplete denitrification phenotypes in diverse Thermus species from diverse geothermal spring sediments and adjacent soils in southwest China
Source: Extremophiles. 2022 Jul 8;26(2):23. doi: 10.1007/s00792-022-01272-1 (PMC9270275; doi:10.1007/s00792-022-01272-1)
Supplement: Supplementary file 1 — Supplementary file1 (DOCX 16 KB) [file 792_2022_1272_MOESM1_ESM.docx]

| Table S1. Primer sequences and positions used to amplify fragments from nitrogen-oxide reductases of *Thermus* strains. | | | |
| --- | --- | --- | --- |
| **Target Gene** | **Primer^a^** | **Position^b^** | **Primer Sequence (5'-3')** |
| ***narG*** | narGn2F_CC | 1013249-1013269 | ACCACCCACGGGGTGAACTGC |
|  | narGn6R_CC | 1012013-1012030 | CTGGGCCATGAGGAGGTC |
|  | narGn7R_CC | 1012025-1012042 | GAGGTCAAAGACGGTGGC |
| ***nirK*** | nirKn3F | 1025543-1025559 | ATGTACCACTGCGCCCC |
|  | nirKn3R | 1024599-1024615 | GGGTGGATGCTGCAGATG |
| ***nirS*** | nirSn1F | 1023891-1023910 | GCCACCCACATCCTGCGCT |
|  | nirSn4R | 1022835-1022857 | GCGGTGTTGTACACGTTGAACTT |
|  | nirSn1FB | 1023891-1023910 | GCCACCCACATCTTGCGCT |
|  | nirSn925RB | 1023408-1023425 | GCGTTGGCCGCCACAATG |
| ***norB*** | norBF1F_CC | 1028097-1028080 | GCCCT**Y**TGGTACTTCTGG |
|  | norBn9R | 1026906-1026927 | GCTCCACCATGGT**Y**TGGGTGAA |
|  | norBn925 (forward) | 1027686-1027703 | CGGTGATGGTTATCTTCC |

a. forward and reverse primers are indicated by the last letters F and R, respectively

b. nucleotide positions in the *Thermus oshimai* JL-2 genome.
