# Supplementary material for: Incomplete denitrification phenotypes in diverse Thermus species from diverse geothermal spring sediments and adjacent soils in southwest China
Source: Extremophiles. 2022 Jul 8;26(2):23. doi: 10.1007/s00792-022-01272-1 (PMC9270275; doi:10.1007/s00792-022-01272-1)
Supplement: Supplementary file 2 — Supplementary file2 (DOCX 14 KB) [file 792_2022_1272_MOESM2_ESM.docx]

Table S2. Primers and annealing temperatures for PCR amplification of denitrification genes.

| **Primer (forward/reverse)** | **Target Gene** | **Annealing Temperature** |
| --- | --- | --- |
| narGn2F_CC/narGn6R_CC | *narG* | 61.3 °C |
| narGn2F_CC/narGn7R_CC | *narG* (strain 318) | 59.6 °C |
| nirKn3F/nirKn3R | *nirK* | 57.2 °C |
| nirSn1F/nirSn4R | *nirS* | 60.9 °C |
| nirSn1FB/nirSn925RB | *nirS* (strain 925) | 64.1 °C |
| norBnF1F_CC/norBn9R | *norB* | 54.9 °C |
| norBn925/norBn9R | *norB* (strain 925) | 55.4 °C |
